# A CRISPR-Cas12a-based platform for ultrasensitive rapid highly specific detection of *Mycobacterium tuberculosis* in clinical application

**DOI:** 10.3389/fcimb.2023.1192134

**Published:** 2023-05-23

**Authors:** Nan Jia, Chaohong Wang, Xiaming Liu, Xiaolan Huang, Fei Xiao, Jin Fu, Chunrong Sun, Zheng Xu, Guirong Wang, Juan Zhou, Yi Wang

**Affiliations:** ^1^ Experimental Research Center, Capital Institute of Pediatrics, Beijing, China; ^2^ Department of Clinical Laboratory, Beijing Chest Hospital, Capital Medical University, Beijing Tuberculosis and Thoracic Tumor Institute, Beijing, China; ^3^ The Second Department of Geriatrics, Handan Central Hospital, Handan, Hebei, China

**Keywords:** *Mycobacterium tuberculosis*, multiple cross displacement amplification, CRISPR, Cas12a, tuberculosis

## Abstract

Tuberculosis, caused by *Mycobacterium tuberculosis* (MTB), is the second leading cause of death after COVID-19 pandemic. Here, we coupled multiple cross displacement amplification (MCDA) technique with CRISPR-Cas12a-based biosensing system to design a novel detection platform for tuberculosis diagnosis, termed MTB-MCDA-CRISPR. MTB-MCDA-CRISPR pre-amplified the specific *sdaA* gene of MTB by MCDA, and the MCDA results were then decoded by CRISPR-Cas12a-based detection, resulting in simple visual fluorescent signal readouts. A set of standard MCDA primers, an engineered CP1 primer, a quenched fluorescent ssDNA reporter, and a gRNA were designed targeting the *sdaA* gene of MTB. The optimal temperature for MCDA pre-amplification is 67°C. The whole experiment process can be completed within one hour, including sputum rapid genomic DNA extraction (15 minutes), MCDA reaction (40 minutes), and CRISPR-Cas12a-gRNA biosensing process (5 minutes). The limit of detection (LoD) of the MTB-MCDA-CRISPR assay is 40 fg per reaction. The MTB-MCDA-CRISPR assay does not cross reaction with non-tuberculosis *mycobacterium* (NTM) strains and other species, validating its specificity. The clinical performance of MTB-MCDA-CRISPR assay was higher than that of the sputum smear microscopy test and comparable to that of Xpert method. In summary, the MTB-MCDA-CRISPR assay is a promising and effective tool for tuberculosis infection diagnosis, surveillance and prevention, especially for point-of-care (POC) test and field deployment in source-limited regions.

## Introduction

Tuberculosis (TB), a substantial global health problem, is a contagious bacterial infection caused by *Mycobacterium tuberculosis* (MTB). It is among the ten top causes of death worldwide and remains a global public health problem. In 2021, there were 10.6 million new diagnosed cases and 1.6 million deaths worldwide (Organization WH, 2022). Recommended TB treatment regimens are lengthy and frequently associated with serious adverse events, which could impact adherence and treatment outcomes (Heyckendorf et al., 2022). Therefore, early diagnosis and screening of TB is critical to limit the spread of the disease, as well as timely treatment.

As the clinical features and radiological findings of MTB infection are not specific, TB diagnosis is mainly based on identification of MTB by smear microscopy for acid fast bacilli (AFB) and culture. Using color-based AFB smear microscope for diagnosis of TB is widely applied in clinical setting, especially in the resource-limited areas, for the merits of simple, rapid and cheap. However, the limited sensitivity and restricted discernibility at species level identification hampered its widely application in accurate detection of MTB. Culture is the traditional “gold standard”, but it relies on lengthy process (4 weeks minimum) and requires strict biosafety conditions (Parsons et al., 2011). Molecular methods has been another powerful tool for MTB identification and has been approved by WHO for TB diagnosis (Pai and Schito, 2015), including GeneXpert MTB/RIF and more. The GeneXpert MTB/RIF assay was endorsed by the WHO in 2010, which could obtain results in two hours. But it had not raised overall detection rates and showed limited efficacy in extrapulmonary tuberculosis diagnosis (Walzl et al., 2018). Thus, simple, rapid and accurate diagnostic methods with high sensitivity and specificity are urgently needed, which is a prerequisite for MTB early and effective treatment and spread control.

Clustered regularly interspaced short palindromic repeat-associated proteins (CRISPR-Cas) is immune system of archaea and bacteria, which could resist the invasion of foreign nucleic acid, such as phage and plasmids (Liu et al., 2022). They have been a popular tool for gene editing, function determining and transcription regulation (Yan et al., 2022). Beyond above, the CRISPR-Cas system has also been employed in biosensing applications for pathogen detection based on the collateral cleavage activity of Cas effectors (such as Cas9, Cas12, and Cas13) (Koonin et al., 2017). The CRISPR-Cas-based biosensing system consists of Cas effector, guide RNA (gRNA) and target double-strand DNA (dsDNA) with an appropriate protospacer-adjacent motif sequence (PAMs) (Chen et al., 2018). When the ribonucleoprotein complex composed of Cas effector and gRNA senses the target dsDNA adjacent to the PAM site and complementary to the gRNA sequence, the cleavage activity of Cas effector was activated, resulting in degradation of the target sequences and the single stranded DNA (ssDNA) reporter labelled with fluorescence and quencher. Dissociation of the reporter can be detected by a fluorescence reader, indicating the presence of target sequences. Although the CRISPR-Cas biosensing system can directly detect nucleic acid targets, the performance will be improved if combined with proper amplification methods. Recently, several CRISPR-Cas-based biosensing paltforms have been established, such as SHERLOCK (Gootenberg et al., 2017), HOLMES (Li et al., 2018), HOLMESv2 (Li et al., 2019) and DETECTR (Chen et al., 2018).

**Figure 1:**
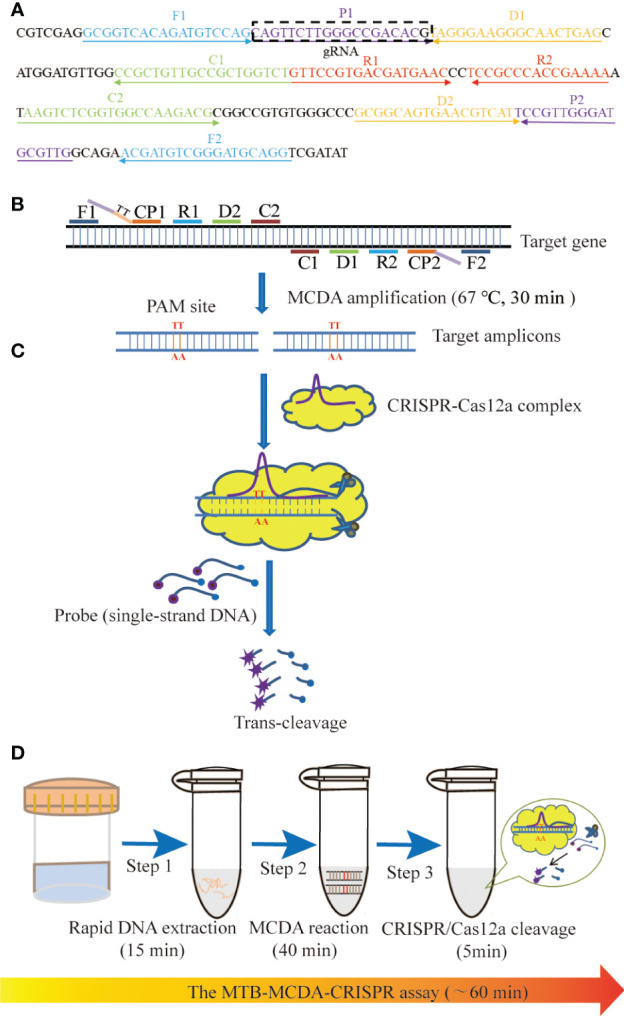
Schematic illustration of the principle of the MTB-MCDA-CRISPR assay. **(A)** Location and sequences of primers and gRNA used in this study. The MCDA primers are positioned with arrows, and the gRNA is in the box. The right and left arrows represent the meaning and complementary sequences used in this study, respectively. **(B)** Schematic diagram of MCDA reaction with the modified primer. Primer CP1 is modified by introducing a PAM site (TT) at the linker region. After amplification, the CRISPR-Cas12a recognition site was constructed in the target amplicons. **(C)** Schematic diagram of CRISPR-Cas12a detection system. When the target DNA is identified, the CRISPR-Cas12a-gRNA complex cuts the single-stranded DNA reporter molecule and releases a fluorescent signal. **(D)** The entire process of the MTB-MCDA-CRISPR detection system. The MTB-MCDA-CRISPR assay is performed by three closely linked steps: DNA extraction (step 1), MCDA pre-amplification (step 2), CRISPR-Cas12a cleavage, and data reporting (step 3), which can be completed within 60 minutes.

In this study, we developed a novel CRISPR-Cas12a-based diagnostic platform termed MTB-MCDA-CRISPR, which combined the CRISPR-Cas12a biosensing system with a promising nucleic acid isothermal amplification technique called multiple cross displacement amplification (MCDA) (Wang et al., 2015) for the timely, accurate, ultra-sensitive and highly specific diagnosis of MTB infection. In addition, due to the lack of proper PAMs within the target sequence, a specific PAMs for Cas12a effector (TT) was introduced into the amplified products by modifying the primers. Here, we elaborated the principle of MTB-MCDA-CRISPR detection (**Figure 1**) and verified its feasibility in clinical specimens.

## Materials and methods

### Reagents and instruments

Genomic DNA kits for nucleic acid extraction and purification were purchased from Beijing TransGen Biotech. Co, Ltd (Beijing, China). The universal isothermal amplification kit was provided from HuiDeXin Biotech. Co., Ltd. (Tianjin, China). EnGen^®^ Lba Cas12a (Cpf1) and 10 × NE Buffer 2.1 were purchased from New England Biolabs (MA, USA). The real-time turbidimeter (LA-320C) was purchased from Eiken Chemical. Co, Ltd (Japan). The ABI7500 real-time fluorescent quantitative PCR systems was purchased from Applied Biosystems (USA). The BluSight Pro (GD50502) was obtained from Manod Biotech. Co, Ltd (Suzhou, China).

### Bacterial strain and clinical samples

All the strains used in this study were shown in **Table 1**, including 8 MTB strains, 9 non-MTB *Mycobacterium* strains and 15 non-*Mycobacterium* strains. All the *Mycobacterium* strains were obtained from Beijing Chest Hospital Affiliated to Capital Medical University (BCH), and the others were from Chinese Center for Disease Control and Prevention (CDC). In addition, a total of 96 sputum samples from 96 patients were also employed in this study. Among them, 77 samples were obtained from TB patients at Beijing Chest Hospital Affiliated to Capital Medical University (BCH), and the other 19 samples were from non-TB patients at Children’s Hospital Affiliated to Center Institute of Pediartic (CIP). The nucleic acid of all the strains and sputum samples were extracted and purified routinely.

**Table 1:** Strains used in this study.

Strains	Source of strains^a^	No. of strains	MTB-MCDA-CRISPR^b^
*Mycobacterium tuberculosis*	Isolated strains (BCH)	8	P
*Mycobaterium cosmeticum*	Isolated strains (BCH)	2	N
*Mycobacterium fortuitum*	Isolated strains (BCH)	1	N
*Mycobaterium kansasii*	Isolated strains (BCH)	1	N
*Mycobaterium abscessus*	Isolated strains (BCH)	1	N
*Mycobaterium gordonae*	Isolated strains (BCH)	1	N
*Mycobaterium komossense*	Isolated strains (BCH)	1	N
*Mycobaterium gadium*	Isolated strains (BCH)	1	N
*Mycobaterium intracellulare*	Isolated strains (BCH)	1	N
*Bacillus cereus*	Isolated strains (CDC)	1	N
*Corynebacterium striatum*	Isolated strains (CDC)	1	N
*Enterococcus faecalis*	Isolated strains (CDC)	1	N
*Escherichia coli*	Isolated strains (CDC)	1	N
*Klebsiella pneumoniae*	Isolated strains (BCH)	1	N
*Listeria innocua*	Isolated strains (CDC)	1	N
*Listeria ivanovii*	Isolated strains(BCH)	1	N
*Monilia albican*	Isolated strains (CDC)	1	N
*Moraxella catarrhalis*	Isolated strains (CDC)	1	N
*Mycoplasma pneumoniae*	Isolated strains (CDC)	1	N
*Neisseria meningitidis*	Isolated strains (CDC)	1	N
*Nocardia asteroides*	Isolated strains (BCH)	2	N
*Pseudomonas aeruginosa*	Isolated strains (CDC)	1	N
*Salmonella* sp.	Isolated strains (CDC)	1	N

a BCH, Beijing Chest Hospital Affiliated to Capital Medical University CDC, Chinese Center for Disease Control and prevetion.

b P, positive; N, negative.

### Multiple cross displacement amplification primers design

A set of ten primers, which targeted the *sdaA* gene of MTB strain H37Rv (Accession no. NC_000962.3), were designed using PRIMER (v5.0). The ten primes were designed based on the MCDA principle and included 2 replacement primers (F1, F2), 2 cross primers (CP1, CP2) and 6 amplification primers (C1, C2, D1, D2, R1, R2). Primers’ specificity were determined by local BLASTN software, and the ones non-specifically matched with other pathogens were excluded. What’s more, according to the CRISPR-Cas12a detection mechanism, a Cas12a-specific PAM site (TT) was introduced into the linker region of CP1 primer, and a gRNA complementary to P1 primer and a probe labelled with FAM and Black Hole Quencher (BHQ1) were designed. All the primers were synthesized by AOKE Biotech. Co., Ltd (Beijing, China), and the gRNA and probe were by Tianyi Huiyuan Biotech. Co., Ltd (Beijing, China). Details of sequences, positions, and modifications of primers, gRNA and probe were shown in **Figure 1A** and **Table 2**.

**Table 2:** Primer sequences used in the study.

Primers	Sequence and modification (5’-3’)^a^	Length^b/c^
F1	GCGGTCACAGATGTCCAG	18 nt
F2	CCTGCATCCCGACATCGT	18 nt
CP1	AGACCAGCGGCAACAGCGG-TT-CAGTTCTTGGGCCGACAC	41 nt
CP2	AAGTCTCGGTGGCCAAGACGCAACGCATCCCAACGGA	37 nt
C1	AGACCAGCGGCAACAGCGG	19 nt
C2	AAGTCTCGGTGGCCAAGACG	20 nt
D1	CTCAGTTGCCCTTCCCTA	18 nt
D2	GCGGCAGTGAACGTCAT	17 nt
R1	GTTCATCGTCACGGAAC	17 nt
R2	TCCGCCCACCGAAAA	15 nt
**gRNA**	UAAUUUCUACUAAGUGUAGAUAGUUCUUGGGCCGACACGUA	41 mer
Probe^c^	FAM-TATTATTATTATTATTT-BHQ1	17 mer

a CP1, primer were modified in the linker region with a PAM site (TT).

b nt, nucleitide.

c mer, monomeric unit.

### Standard multiple cross displacement amplification assay

The standard MCDA reaction was performed in a 25 μL reaction mixture, including 12.5 μL of 2× reaction buffer, 0.4 μM each of F1 and F2, 1.6 μM each of CP1 and CP2, 0.8 μM each of C1, C2, D1, D2, R1 and R2, 1.0 μL of *Bst 2.0* DNA polymerase (8 U), 1 μL (pure bacteria) or 5μL (clinical sample) DNA template, and supplemented with distilled water to 25 µL. The amplification results were monitor by the real-time turbidity (LA-320C). Besides, in order to optimize the whole detection procedure, the optimal reaction temperature during pre-amplification stage was further determined by conducting MCDA reaction at temperatures ranging from 62 to 69°C at 1°C intervals. DNA template of MTB strain H37Rv was used as positive control, that of *M. cosmeticum* as negative control, and distilled water (DW) as blank control. Each test was performed at least three times.

### CRISPR-Cas12a-based detection

CRISPR-Cas12a-based biosensing system was used to detect the pre-amplified products of MCDA assay on the basis of the trans-cleavage activity of Cas12a effector. Firstly, a complex composed of Cas12a effector and gRNA complementary to the target sequence was prepared by mixing 100 nM Cas12a effector with 100 nM gRNA in 2×HDX buffer solution at 37°C for 10 min. The complex should be used immediately or stored at a low temperature (0-4°C) for no more than 12 hours. Then, the CRISPR-Cas12a trans-cleavage assay was conducted in a 100 μL mixture, which included 18 μL CRISPR-Cas12a-gRNA complex, 2 μL MCDA product, 2.5 μL probe, 50 μL 2×HDX buffer, and 27.5 μL distilled water. The detection mixture was incubated at 37°C for 10 min on a real-time PCR platform for result reporting. In addition, the resulting fluorescence signals were also visually detected by the naked eye under blue light. The probe used in CRISPR-Cas12a trans-cleavage was 5’- FAM-TATTATTATTATTATTT-BHQ1-3’ (10 μM).

### Sensitivity and specificity of MTB-MCDA-CRISPR method

To verify the specificity of the MTB-MCDA-CRISPR assay, a total of 8 MTB strains and 24 non-MTB strains were employed in this study (**Table 1**). For the sensitivity analysis, genomic DNA templates of the MTB strain H37Rv were ten-fold serially diluted from 4 ng to 4 fg per microliter and subjected to MTB-MCDA-CRISPR test, with DW as the blank control. Each test was conducted three replicates. All the results were analyzed by both the real-time PCR platform and naked eyes under blue light.

### Verification of clinical feasibility of MTB-MCDA-CRISPR method

To evaluate the feasibility of MTB-MCDA-CRISPR detection method in clinical settings, a total of 96 sputum specimens were employed, 77 of which were collected from TB patients, and 19 were from non-TB patients. All the samples were tested by MTB-MCDA-CRISPR assay, sputum smear microscopy test and Xpert assay simultaneously. The performance of the MTB-MCDA-CRISPR assay was compared with that of the other two methods.

## Results

### Schematic mechanism of the MTB-MCDA-CRISPR detection system

**Figure 2:**
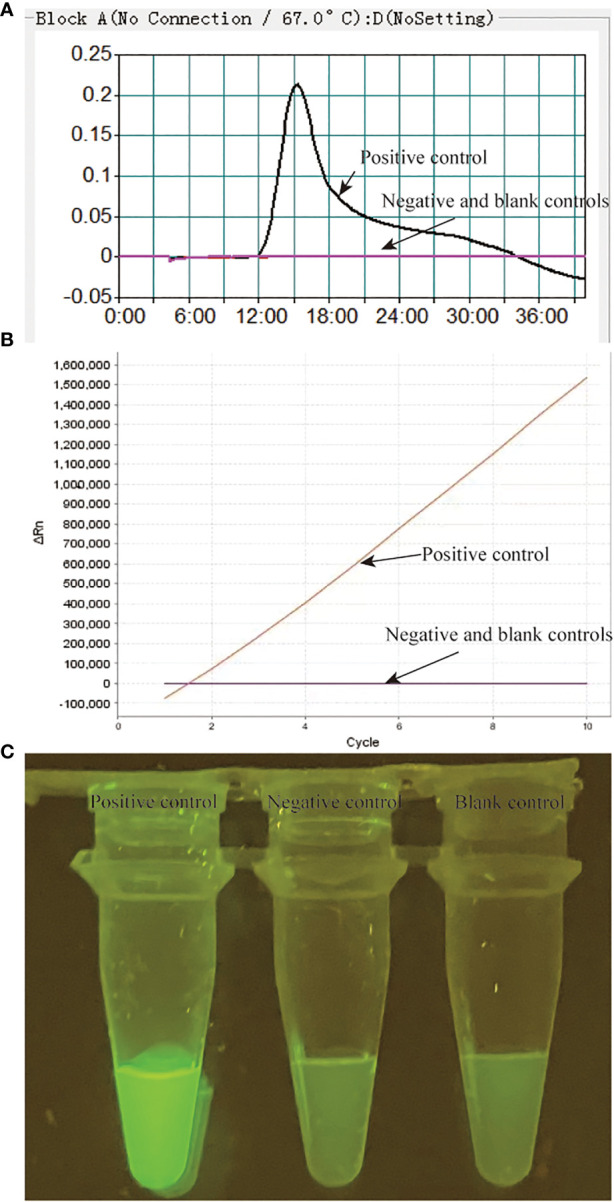
Validation of the MTB-MCDA-CRISPR assay for detection of MTB. The results of the MTB-MCDA-CRISPR assay was assessed by real-time turbidity detection **(A)**, real-time fluorescence detection **(B)** and visual detection by naked eye under blue light **(C)**. Genomic DNA of MTB strain H37Rv was used as positive control, that of the *Mycobaterium cosmeticum* was as negative control and distilled water was as blank control.

As shown in **Figure 1**, the MTB-CDA-CRISPR detection system was established based on the isothermal amplification method MCDA and the “ssDNA collateral effect” of Cas12a effector. In the MTB-MCDA-CRISPR detection system, MCDA approach was employed for rapidly and specifically amplifying target sequences with fixed conditions (67°C, 40 min) (**Figure 1B**). The principle of MCDA method has been reported in previous study (Wang et al., 2015; Li et al., 2020). Moreover, the core primer CP1 in this study was modified by adding a PAM site (TT) in its linker region. Thus, a plenty of amplicons containing PAM site specific for Cas12a effector were produced, which can be recognized by the CRISPR-Cas12a-gRNA binary complex. Once there exist target DNA amplicons in the reaction mixture, the CRISPR-Cas12a-gRNA binary complex would form a ternary complex with the target DNA amplicons, which will activate the trans-cleavage activity of Cas12a effector, resulting in non-targeted ssDNA reporter trans-cleaved and fluorescence signals illuminated **(** **Figure 1C**). The fluorescence signals can be visualized by Applied Biosystems^®^7500 Real Time PCR system or by naked eyes under blue light with BluSight Pro system. No fluorescence signal can be detected if no target amplicons exist, implying a negative result. As shown in **Figure 2**, the positive control reaction mixture displayed distinct turbidity increase (**Figure 2A**), remarkable fluorescence values on real-time PCR platform (**Figure 2B**) and visible fluorescence signals under blue light (**Figure 2C**), while the negative and blank controls did not show any turbidity or fluorescence values/signals. These results confirmed that the MTB-MCDA-CRISPR detection system was able to amplify target MTB DNA sequences and detect them based on the CRISPR biosensing system with real-time PCR apparatus or by naked eye under blue light.

### Optimal reaction temperature and time of MTB-MCDA-CRISPR method

Performance of the MTB-MCDA assay at temperatures ranging from 62-69°C was shown in **Figure S1**. It showed that the optimum reaction temperature for MTB-MCDA step of this study was 67°C, at which a fastest and most productive amplification result was obtained.

By comparing the trans-cleavage efficiency of CRISPR-Cas12a effector at a reaction time of 5, 10, and 15 min, respectively, we found that a least trans-cleavage time of 5 min was enough for CRISPR-Cas12a effector to recognize the target DNA amplicons and trans-cleave the ssDNA reporters (**Figure S2**). Thus, the MTB-MCDA-CRIPSR detection system was able to detect MTB within 1 h, including 15 min for rapid DNA extraction, 40 min for MCDA pre-amplification and 5 min for result reporting (**Figure 1D**). The optimal reaction temperature and time were therefore used in all the subsequent tests.

### Specificity and sensitivity of MTB-MCDA-CRISPR method

**Figure 3:**
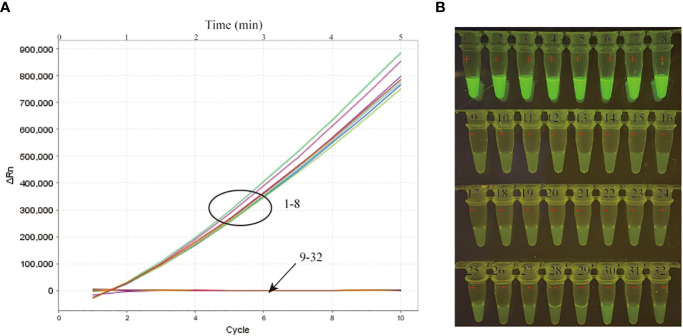
Specificity verification of the MTB-MCDA-CRISPR detection system. A total of 8 MTB strains (signals/tubes 1-8) and 24 non-MTB strains (signals/tubes 9-32) were tested by the MTB-MCDA-CRISPR assay for specificity evaluation. The produced fluorescent signals were document in a real-time manner by the real-time PCR system **(A)** and visually interpreted by naked eye under blue light **(B)**.

A specificity evaluation was performed by using various templates extracted from MTB and other bacteria (**Table 1**). Among all the strains tested in our study, the positive results were only obtained from MTB strains (**Table 1**; **Figure 3**). In addition, no cross-reaction to other bacteria strains were produced in our study. Thus, our data indicated that the MTB-MCDA-CRISPR assay was highly specific (100%) to detect the MTB.

**Figure 4:**
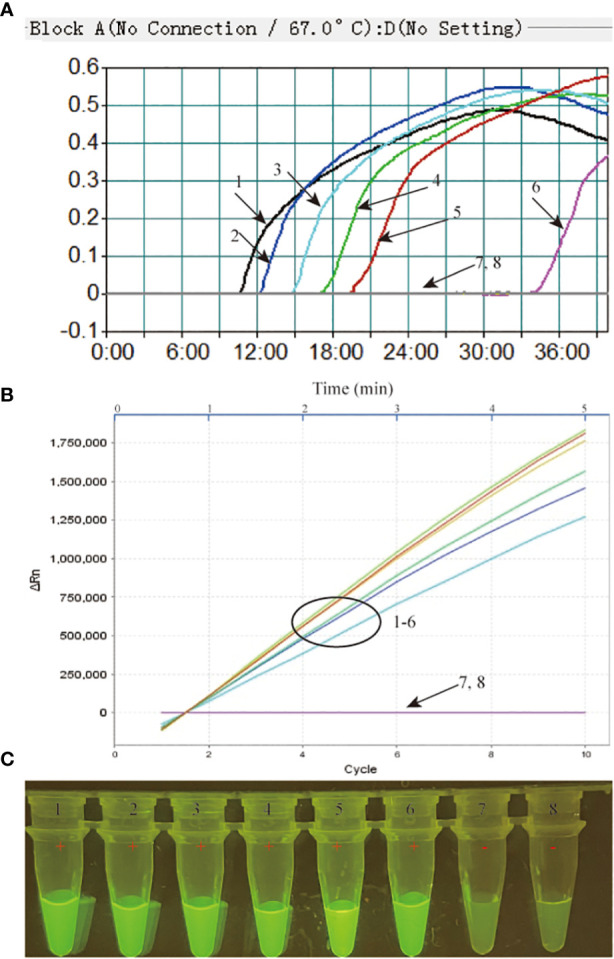
Sensitivity assessment of the MTB-MCDA-CRISPR detection system. The genomic DNA of MTB strain H37Rv was ten-fold serially diluted from 4 ng to 4 fg per microlitre and used as templates for sensitivity analysis, with distilled water (DW) as blank control. All the detection results were recorded by measuring the real-time turbidity **(A)**, real-time fluorescence intensity **(B)** and visually interpreted by naked eye under blue light **(C)**. Signals/tubes 1-7 represent the results of the MTB genomic DNA of 4 ng, 400 pg, 40 pg, 4 pg, 400 fg, 40 fg and 4 fg, respectively, and signal/tube 8 represent the result of bank control.

The limit of detection (LoD) of our MTB-MCDA-CRISPR assay was investigated using various dilutions of genomic templates taken from pure cultures of MTB H37Rv (4 ng, 400 pg, 40 pg, 4 pg, 400 fg, 40 fg and 4 fg per microliter). As shown in **Figure 4**, the results by real-time PCR platform and by naked-eye under blue light were nearly identical, all indicating that the MTB-MCDA-CRISPR assay can detect low to 40 fg of MTB genomic DNA per reaction.

### Feasibility validation of MTB-MCDA-CRISPR method in clinical samples

**Figure 5:**
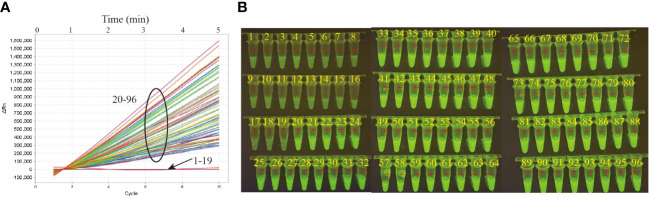
Performance of the MTB-MCDA-CRISPR detection system in clinical samples. A total of 96 samples were tested by the MTB-MCDA-CRISPR assay to validate the clinical feasibility. The generated fluorescence signals were recorded by the real-time PCR platform **(A)** and visually interpreted by naked eye under blue light **(B)**. Signals/tubes 1-19 present the results of the 19 samples from non-TB patients, and Signals/tubes 20-96 represent the results of the 77 samples from TB patients.

**Figure 6:**
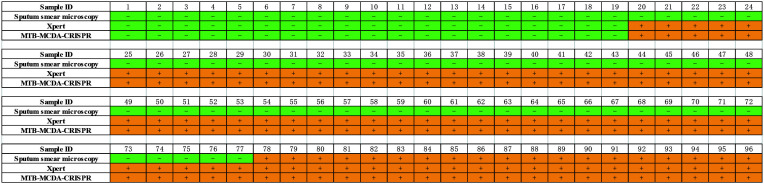
Comparison of the MTB-MCDA-CRISPR assay, Xpert assays and sputum smear microscope test among the clinical sample for both TB and non-TB patients.

In order to validate the clinical feasibility of MTB-MCDA-CRISPR assay, we examined DNA templates extracted from 77 MTB-positive sputum samples and 19 non-MTB infection sputum samples. All the sputum samples had been diagnosed by both sputum smear microscopy and Xpert methods previously. Among the 77 MTB-positive sputum samples, 19 were positive for both sputum smear microscopy and Xpert tests, and 58 were positive for Xpert test but negative sputum smear microscopy test. All the 19 non-MTB infection sputum samples were negative for both sputum smear microscopy and Xpert tests. As shown in **Figure 5**, the MTB-MCDA-CRISPR assay identified all the 77 MTB-positive sputum samples, including the 58 samples negative for sputum smear microscopy test; no positive result was observed in the 19 non-MTB infection sputum samples. The detection results of MTB-MCDA-CRISPR assay were identical to those of the Xpert method, but better than those of sputum smear microscopy method (**Figure 6**). These results indicated that the MTB-MCDA-CRISPR assay can be used as an advanced technology to detect the MTB infection in clinical settings.

## Discussion

Tuberculosis is a chronic infectious disease worldwide. Early and accurate diagnosis of tuberculosis is essential to achieve global tuberculosis control (Furin, 2019). Traditional bacteria-based methods (such as sputum smear microscopy and MTB bacilli culture) were sensitive-limit and time-consuming. Thus, nucleic acid-based diagnostic tools for MTB detection were still urgently needed. In this study, we established a diagnostic method called MTB-MCDA-CRISPR, which takes the advantage of MCDA and CRISPR-cas12a system, and has been demonstrated to confer high sensitivity and specificity.

Nucleic acid detection is crucial for numerous applications such as molecular biology research and medical diagnosis (Obande and Banga Singh, 2020). Recently, several nucleic acid detection methods have been established and approved by WHO for diagnosing MTB infection, such as Xpert and TB-LAMP (loop-mediated isothermal amplification) (Organization WH, 2013; Organization WH, 2016). Xpert was a polymerase chain reaction (PCR)-based MTB detection platform and had exhibited great potential in MTB diagnosis owning to its high sensitivity and specificity (Horne et al., 2019). However, this kind of diagnostic platform was highly depended on specialized laboratory instrument and personnel and high cost, which hampered the widely application in the MTB-high-burden areas. TB-LAMP was a simple, rapid, user-friendly and sensitive diagnostic tool that developed based on isothermal amplification technique and bypassed the shortcomings of Xpert-associated platforms (Sam et al., 2021). Recently, several methods combining LAMP technique and different detection system, such as lateral flow biosensors (Yang et al., 2021) and CRISPR system (Ai et al., 2019; Sam et al., 2021; Yang et al., 2021), have been developed for easy and accurate detection of MTB. In addition, several other isothermal amplification techniques have been employed in MTB identification as well, such as recombinase polymerase amplification (RPA) (Boyle et al., 2014) and MCDA (Jiao et al., 2019). Of note, combination of nucleic acid amplification technique and CRISPR system could improves both detection sensitivity and accuracy. Thus, in this study, we integrated MCDA for MTB DNA pre-amplification with CRSIPR-Cas12a biosensing system for products detection to further develop a simple, rapid, affordable and accurate diagnostic tool for MTB infection, which could be performed only depend on a simple water bath for pre-amplification and a blue light instrument for result report by naked eye, consisting with the principles of point-of-care test (POCT).

In this study, the MCDA method was employed for MTB nucleic acid amplification. Only with a simple container that can sustain a constant temperature (67 °C), products of the target gene *sdaA* of MTB were exponential produced in a short time (40 min). In addition, the primers covered ten different regions of the target sequence, ensuring the high specificity of the amplicons. Moreover, although no available PAM site exists within the target sequence for subsequent CRISPR-Cas12a-based detection, we introduced a PAM site (TT) at the linker region of the primer CP1, resulting in production of a plenty of amplicons including PAM site, which enable the MTB-MCDA-CRISPR detection platform to work in a PAM independent way. After MCDA reaction, the CRISPR-Cas12a-based biosensing system was utilized to test the presence or absence of target sequence. Under the guidance of gRNA complementary to the target sequence and recognition of PAM site, the pre-amplified amplicons were retargeted and trans-cleaved by the Cas12a effector, along with the collateral cleavage of ssDNA reporters which were labelled with paired fluorescence/quencher (FAM-TATTATTATTATTATTTT-BHQ1), generating detectable fluorescent signal. The generated fluorescent signals could be detected not only by a real-time RCR platform but also by naked eye under blue light within only 5 min. The further enhanced specificity, ultra-sensitivity, easy to use and speed of CRISPR-Cas12a biosensing system facilitated the performance of MTB-MCDA assay greatly boosted. Thus, the whole process for MTB infection diagnosis, from nucleic acid extraction to result reporting, can be completed within 1h (**Figure 1D**).

The MTB-MCDA-CRISPR assay developed here was proved highly specific for MTB identification. In this study, only the 8 MTB strains were tested positive for the MTB-MCDA-CRISPR assay, while the other pathogens including the 9 NTM (non-tuberculous mycobacteria) strains were all negative for this assay. The elimination of cross-activity with other pathogens of the MTB-MCDA-CRISPR detection system ensures its reliability in MTB infection diagnosis, which was apparently superior to the sputum smear microscopy method that only can identify at genus level.

Sensitivity analysis demonstrated that the MTB-MCDA-CRISPR assay targeting *sdaA* gene was ultra-sensitive to diagnose MTB infection. The MTB-MCDA-CRISPR assay was able to determine as low as 40 fg (~8 copies) of MTB genome DNA per reaction, which was more sensitive than the previously devised MCDA-LFB assay targeting the *IS6110* and *mpb64* genes (100 fg) (Huang et al., 2021) and the Xpert MTB/RIF Ultra detection platform (15.6 cfu/ml) (Organization WH, 2017), comparable to the performance of TB-QUICK platform (1.3 copy/μL MTB DNA) (Sam et al., 2021). The low detection limit of the MTB-MCDA-CRISPR assay enables its application in bacilli low-burden TB patients.

When the significance of the MTB-MCDA-CRISPR assay was evaluated in clinically derived specimens, we found that the MTB-MCDA-CRISPR assay was feasible in the clinic. Identical to the Xpert test, the MTB-MCDA-CRISPR assay identified all the positive samples, no matter sputum smear microscopy test positive or negative, illustrating a high sensitivity and detection efficiency. Besides, all the negative clinical samples were tested negative by the MTB-MCDA-CRISPR assay as well, further revealing its high degree of specificity for clinical diagnosis. Of note, our new devised MTB-MCDA-CRISPR assay correctly discerned all the sputum smear microscopy test negative TB patient samples, further emphasizing the advantages of our assay in paucibacillary patients. Recently, besides the novel detection platform devised here, several CRISPR-based assays for MTB detection, including the TB-QUICK detection platform (Sam et al., 2021), the LACD detection platform (Wang et al., 2021), the CRISPR-MCDA system and more (Yang et al., 2023), also have been developed for better diagnosing and controlling of MTB infection, all of which have been validated with excellent performance in clinical practices, further illustrating the importance of diagnosis of MTB with rapidity and accuracy for public health. Given the limited amount of sample size, the efficiency of our MTB-MCDA-CRISPR detection system for clinical practice need to be further verified. Moreover, in this study, detection of MTB required a step of opening of the reaction tube, which increased the risk of carryover contamination in the laboratory. Hence, the risk of carryover contamination also should be evaluated and surmounted in the further, including a one-step detection procedure being devised.

Optimum POC test for rapid diagnosis of TB and latent MTB infection needs to satisfy the characteristics of accuracy, affordability, rapidity, easy-to-use, sensitivity and specificity and can be performed without complicated instruments or specialized personnel (Uplekar et al., 2015). In the new developed MTB-MCDA-CRISPR detection platform here, only a simple water bath and a portable blue light instrument are needed, and the colorimetric fluorescent readout can be interpreted visually by naked eye, all of which are well suited for the POC test and field deployment diagnostics that extremely desired in resource-limited regions and first-line laboratories. Therefore, the MTB-MCDA-CRISPR detection platform developed in this study was a promising diagnostic tool for detection, surveillance and prevention of MTB infections.

## Conclusion

Herein, we have developed a new detection method called MTB-MCDA-CRISPR by combining the MCDA with CRISPR-Cas12a system. MTB-MCDA-CRISPR assay can detect MTB genomic DNA as low as 40 fg per reaction, and does not cross-react with other pathogens. The entire testing procedure can be completed in 60 minutes without complicated instruments or experienced technicians. Moreover, this approach has been demonstrated to confer high sensitivity and specificity in clinical settings. Thus, the newly developed MTB-MCDA-CRISPR assay is expected to become an important method for MTB infection diagnosis.

## Data availability statement

The original contributions presented in the study are included in the article/**Supplementary Material**. Further inquiries can be directed to the corresponding authors.

## Ethics statement

The studies involving human participants were reviewed and approved by Beijing Chest Hospital Ethics Committee (Ethical approval no. KY-2018-020).

## Author contributions

NJ performed the experiments, analyzed the data and drafted the manuscript. CW and XL performed the experiments, provided reagents and materials. CS, FX, JF, XH, and ZX provided reagents and materials. JZ and GW supervised this study and revised the manuscript. YW conceived, supervised and funded this study as well as revised manuscript. All authors contributed to the article and approved the submitted version.

## Supplementary material

Supplementary Figure 1 Screening of MTB-MCDA reaction temperature for MTB detection. Optimization of reaction temperature of the MTB-MCDA assay was conducted by performing the MTB-MCDA assay at temperatures ranging from 62 to 69 °C with 40 pg of MTB genomic DNA as template, with the distilled water as blank control. The amplification efficiency at different temperatures was evaluated; the real-time turbidity values at the eight temperatures for 40 min of reaction are shown in kinetic graphs **(A–H)**.

Supplementary Figure 2 Screening of CRISPR-Cas12a trans-cleavage time for MTB detection. Optimization of CRISPR-Cas12a trans-cleavage time was implemented by recording the performance at different reaction time: the fluorescence values at a reaction time of 5 min, 10 min and 15 min, respectively, were documented in a real-time manner by the real-time PCR system **(A–C)** and visually interpreted by naked eye under blue light **(D)**. Signals/tubes 1-7 represent the results of the MTB genomic DNA of 4 ng, 400 pg, 40 pg, 4 pg, 400 fg, 40 fg and 4 fg, respectively, and signal/tube 8 represent the result of bank control.
