# Supplementary figures and images for: A CRISPR-Cas12a-based platform for ultrasensitive rapid highly specific detection of Mycobacterium tuberculosis in clinical application
Source: Front Cell Infect Microbiol. 2023 May 23;13:1192134. doi: 10.3389/fcimb.2023.1192134 (PMC10242030; doi:10.3389/fcimb.2023.1192134)

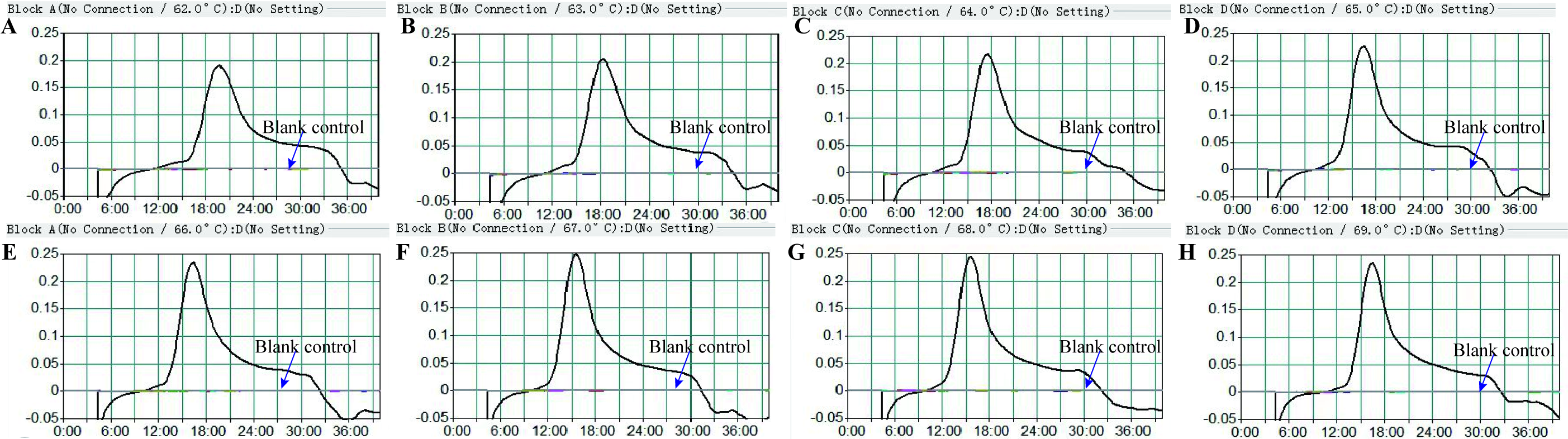

Supplement: Supplementary Figure 1 — Screening of MTB-MCDA reaction temperature for MTB detection. Optimization of reaction temperature of the MTB-MCDA assay was conducted by performing the MTB-MCDA assay at temperatures ranging from 62 to 69 °C with 40 pg of MTB genomic DNA as template, with the distilled water as blank control. The amplification efficiency at different temperatures was evaluated; the real-time turbidity values at the eight temperatures for 40 min of reaction are shown in kinetic graphs (A–H). [file Image_1.tif]

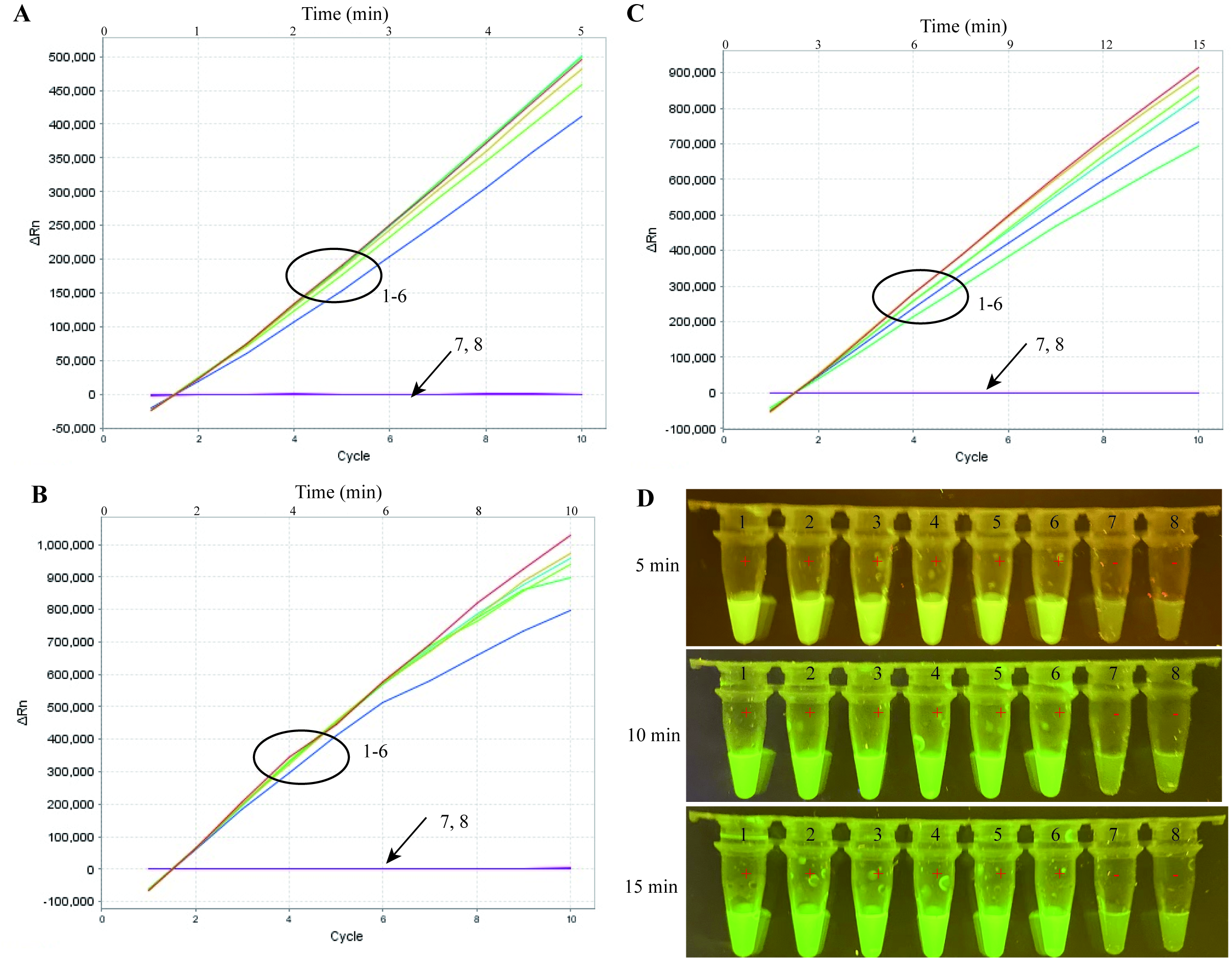

Supplement: Supplementary Figure 2 — Screening of CRISPR-Cas12a trans-cleavage time for MTB detection. Optimization of CRISPR-Cas12a trans-cleavage time was implemented by recording the performance at different reaction time: the fluorescence values at a reaction time of 5 min, 10 min and 15 min, respectively, were documented in a real-time manner by the real-time PCR system (A–C) and visually interpreted by naked eye under blue light (D). Signals/tubes 1-7 represent the results of the MTB genomic DNA of 4 ng, 400 pg, 40 pg, 4 pg, 400 fg, 40 fg and 4 fg, respectively, and signal/tube 8 represent the result of bank control. [file Image_2.tif]
